# Can Farmed and Wild Salmon Coexist?

**DOI:** 10.1371/journal.pbio.0060046

**Published:** 2008-02-12

**Authors:** Liza Gross

When the cod industry off Canada's eastern shore collapsed in 1992, industrial fisheries could no longer ignore biologists' warnings that the ocean would not sustain unlimited exploitation of its fish stocks. But a nearly 5-fold increase in global marine catch over four decades had already taken its toll, placing the persistence of scores of the sea's giant predators—long-lived species that take several years to reach sexual maturity, like bluefin tuna, halibut, and sharks—in serious jeopardy. Many organizations, including the World Bank, saw aquaculture (raising fish in ponds or open net pens in bays) as the best way to relieve pressure on depleted wild populations while meeting consumer demand. Aquaculture now accounts for over 30% of the world's fish market and about half of store-bought salmon.

Yet the solution once hailed as a panacea has come under increasing fire for polluting coastal waters and threatening wild populations. And now, a new study by Jennifer Ford and Ransom Myers reports that salmon farming's impacts on wild salmonid populations—which have undergone drastic declines in the North Atlantic and northeastern Pacific since the late 1980s—are even worse than feared.

Some of aquaculture's problems have been known for years. Farming carnivorous fish like salmon depletes wild stocks of other species—on average, every pound of farmed salmon consumes three pounds of wild-caught fish. Fish farms foul coastal waters with a long list of organic and chemical contaminants, including feces that choke marine life with excess nutrients, surplus additive-laden feed, antibiotics, pesticides, toxic antifouling paints, and disinfectants. And for a species that in the wild can swim hundreds of kilometers against upstream currents, often scaling waterfalls, to return from the ocean to natal spawning grounds, open net pens pose little barrier to escape, and millions do each year. Escapees may reduce survival rates of wild populations—a major concern for Atlantic salmon, the most popular farmed salmon species—by competing for mates and diluting the genetic makeup of their wild counterparts through hybridization.

A study published in December (http://www.sciencemag.org/cgi/content/abstract/318/5857/1772), coauthored by Ford and Myers, found that wild pink salmon populations north of Vancouver Island suffered recurrent louse infestations and population declines in association with salmon farms. In their new study, Ford and Myers analyze the impacts of salmon farms around the globe and report that farms dramatically reduce survival rates of wild salmonids that migrate past aquaculture operations as juveniles on their way to the ocean. Myers, whose death last March claimed one of the most powerful voices for marine conservation, developed groundbreaking analytical methods to show that industrial fisheries caused the cod collapse and later revealed a catastrophic decline of the ocean's large predatory fish.

Many studies have examined the effects of a single aspect of salmon farming—for example, disease transmission or hybridization—but few have investigated the collective impacts of aquaculture on wild populations. To detect population level trends over time, Ford and Myers gathered as much data on survival and abundance as possible from published and unpublished sources (using an approach Myers pioneered) for five salmonid species—Atlantic salmon; sea trout; and pink, chum, and coho salmon—in regions where both farmed and wild salmon occur—Ireland, Wales, and Scotland, and three regions in Canada: Newfoundland, New Brunswick, and British Columbia. The authors developed mathematical models to estimate changes in abundance and survival of wild salmonids that breed near farms and compared these trends with those estimated for unexposed populations. They considered a population to be exposed if its river spawning grounds discharged either into bays or channels with at least one farm or into bays near areas with several farms, increasing the likelihood that juveniles would swim by a farm. Populations were considered unexposed, and served as controls, if their migratory route made it highly unlikely that young fish would pass a farm. Confounding effects of variable climate and anthropogenic influences were reduced by pairing exposed and control populations from the same region.

In most paired comparisons, salmon farming reduced the likelihood that the wild fish would survive or return to their natal spawning grounds. In many cases, survival and returns dropped by over 50% per generation. If all the wild populations migrated past farms averaging a 15,000-tonne annual yield, survival rates dropped by 73% on average—a sobering result given that production in many of these regions exceeds 20,000 tonnes per year, and shows no signs of abating.

Atlantic salmon (and Irish sea trout) populations suffered greater declines than Pacific salmon did, possibly because wild and farmed Atlantic salmon can interbreed, adding any deleterious genetic effects to other potential impacts. Though Irish sea trout cannot interbreed with salmon, they spend more time in coastal waters than other species, which could make them more susceptible to transmitted parasites and disease. British Columbia pink salmon also showed more substantial declines linked to salmon farming.

Salmon farming's impacts did not increase linearly with salmon production, the authors point out, a trend that could reflect improvements in aquaculture management. (Their datasets go back to aquaculture's origins in each region.) But the rapid growth of aquaculture—the industry accounted for 33% of global food production in 2000 (about 40 million tonnes, worth US$55 billion) and is expected to claim 50% of the market in the next decade (over 100 million tonnes, worth over US$150 billion)—suggests that any improvements will lag far behind the expansion.

Given that salmon farming could seriously compromise the persistence of the world's salmonid populations, what do the inheritors of Myers' legacy recommend? Though some environmental organizations want to eliminate salmon farming altogether, removing aquaculture operations from the migratory path of wild juveniles—or not placing them along migration corridors to begin with—would greatly improve the survival of wild populations. Some countries, including Iceland and Norway, already have these protections in place. In addition, closed containment could substantially reduce not only the threat of genetic introgression from farmed to wild salmon but also the transmission of most diseases. Until then, consumers can do their part by choosing seafood wisely. Some nonprofits, including The Marine Stewardship Council (http://eng.msc.org/), run certification programs and issue guides to help consumers choose sustainable seafood—and ensure that wild salmon continue to make their improbable journey from stream to sea and back again, as they have done for millions of years.

**Figure:**
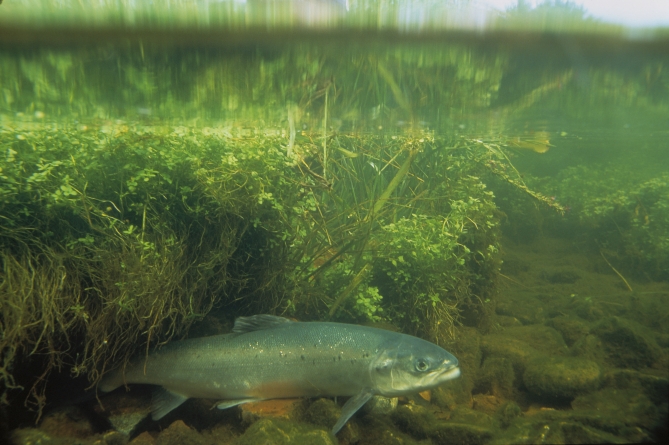
A global survey of wild salmon and trout populations reveals substantially reduced survival among those populations that migrate past salmon farms as juveniles on their way to the ocean. Above: Atlantic salmon. (Image: United States Fish and Wildlife Service)
